# Anticancer Activity of *Garcinia morella* on T-Cell Murine Lymphoma Via Apoptotic Induction

**DOI:** 10.3389/fphar.2016.00003

**Published:** 2016-01-29

**Authors:** Bhaswati Choudhury, Raghuram Kandimalla, Rupjyoti Bharali, Javadi Monisha, Ajaikumar B. Kunnumakara, Kasturi Kalita, Jibon Kotoky

**Affiliations:** ^1^Drug Discovery Laboratory, Life Sciences Division , Institute of Advanced Study in Science and TechnologyGuwahati, India; ^2^Department of Biotechnology, Gauhati UniversityGuwahati, India; ^3^Cancer Biology Laboratory, Department of Bioscience and Bioengineering, Indian Institute of TechnologyGuwahati, India; ^4^Department of Pathology, Hayat HospitalGuwahati, India

**Keywords:** Dalton’s lymphoma, antioxidant, cytotoxicity, apoptosis, caspase3

## Abstract

Traditional knowledge (TK) based medicines have gained worldwide attention and presently the scientific community is focussing on proper pharmacological validation and identification of lead compounds for the treatment of various diseases. The North East region of India is the home of valuable traditional herbal remedies. *Garcinia morella* Desr. (Guttiferae) is one such medicinal plant used by traditional healers for the treatment of inflammatory disorders. The present study was aimed to evaluate the antioxidant and anticancer activity of methanol extracts of the leaf, bark and fruit of *G. morella* (GM) in different *in vitro* and *in vivo* experimental conditions. The results of this study showed that GM methanol extracts possessed *in vitro* antioxidant and anticancer properties, where the fruit extract (GF) showed maximum activity. The anticancer activity was further confirmed by the results of *in vivo* administration of GF (200 mg/kg) for ten days to Dalton’s lymphoma (DLA) induced mice. GF extract significantly increased the mean survival time (MST) of the animals, decreased the tumor volume and restored the hematological and biochemical parameters. The present study for the first time reported the anticancer property of GF on DLA. Further from the experiments conducted to elucidate the mechanism of action of GF on DLA, it can be concluded that GF exerts its anticancer effect through induction of caspases and DNA fragmentation that ultimately leads to apoptosis. However, further experimentation is required to elucidate the active principle and validate these findings in various *in vivo* settings.

## Introduction

Cancer is a leading cause of death worldwide accounting for 8.2 million deaths in 2012 (Ferlay et al., 2015). The American Cancer Society estimates that around one and half million new cancer cases are expected to be diagnosed Siegel et al. (2015) in the United States alone. Lymphomas are a type of cancer that affects the bone marrow, blood cells, lymph nodes, and other parts of the lymphatic system. According to GLOBACON 2012 (WHO), non-hodgkin’s lymphoma is one of the ten most diagnosed cancers in men around the world.

Chemotherapy is the common treatment for cancer, but often it is associated with poor quality of life for the cancer patients, primarily due to treatment related side effects (Lotfi-Jam et al., 2008). To minimize the side effects of the synthetic drugs, the scientific community is now focussing largely on natural product derived drugs (Kunnumakara et al., 2005; Kuete and Efferth, 2010). So far, more than 25,000 different phytochemicals have been identified that may have potential against various cancers (Anand et al., 2008). More than 25% of the drugs that have come in market during the last 20 years are plant-derived (Amin et al., 2009) some of the notable ones being vincristine, vinblastine, etoposide, paclitaxel, curcumin, and campothecine. In India, traditional medicine systems continue to be widely prevalent. Chemical investigation of Indian medicinal plants has the potential to lay an invaluable foundation for the discovery and development of new drugs of natural origin (Balunas and Kinghorn, 2005). However, evidence based determination of the pharmacological properties of many such medicinal plants remains poorly studied. Scientific validation of medicinal plants used by traditional practioners is a vital part of ethnopharmacology (Shajib et al., 2015).

*Garcinia morella* Desr. belongs to Guttiferae family and is a large genus of polygamous trees or shrubs, distributed in the tropical Asia, Africa and Polynesia. *Garcinia* species are a rich source of secondary metabolites including xanthones, flavonoids, benzophenones, lactones, and phenolic acids (Gustafson et al., 1992). The *Garcinia* species of plants are well known for their antibacterial (Rao and Verma, 1951; Sani and Rao, 1969) anti-inflammatory, antioxidative (Yamaguchi et al., 2000) antifungal activities etc (Gopalakrishnan et al., 1997). Xanthones and prenylated benzophenones isolated from *Garcinia* have shown to be cytotoxic (Mackeen et al., 2000; Huang et al., 2011; Wang et al., 2012; Li et al., 2013a) against different human cancer cell lines in both *in vitro* and *in vivo* studies. Chemical constituents like morelloflavone, guttiferic acid, and gambogic acid were reported from *G. morella* plant (Karanjgaokar et al., 1967; Rajagopal Rao et al., 2007; Chantarasriwong et al., 2010). *G. morella* locally known as kuji thekera is a medicinal plant used by the locals and tribal people of the north eastern region of India to cure stomach ailments, bowel disorders and inflammatory diseases. However, there is no scientific validation of *G. morella* fruits being used against Dalton’s lymphoma ascites tumour (DLA). DLA is a T-cell lymphoma originating spontaneously in the thymus of DBA (H2^D^) strain of mice (Klein, 1951; Deepak et al., 2007). It has been noticed that spontaneously arising animal tumors mimic a situation more similar to human neoplasia than experimentally induced tumors (Ben-Efraim et al., 1999). Therefore, we aimed to evaluate the antioxidant and anticancer activities of *G. morella* (GM) methanol extracts against DLA tumor models and elucidate the probable molecular mechanism of action.

## Materials and Methods

### Chemicals and Reagents

2,2-Diphenyl-picrylhydrazyl (DPPH), butylated hydroxyl toluene (BHT), tris (hydroxymethyl) aminomethane (Tris), ethylenediamine tetraacetic acid (EDTA), potassium ferricyanide, trichloroacetic acid, thiobarbituric acid, trypan blue, propidium iodide, 3-(4,5-dimethylthiozol-2-yl)-2,5 diphenyltetrazolium bromide dye (MTT) were purchased from Sigma–Aldrich., USA. Aspartate transaminase (AST), Alanine aminotransferase (ALT), and Alkaline phosphatase (ALP) kits were purchased from Accurex, India. Remaining chemicals used in this study were analytical grade and procured either from Sigma–Aldrich, USA or Merck, Germany.

### Plant Collection and Identification

Fresh fruit, leaves, and bark of *G. morella* were collected from Barpeta district of Assam, India in the month of February. Plant materials were authenticated by taxonomist at North East Indian Ayurvedic Research Institute (Government of India) Guwahati, Assam, India. Herbarium was prepared and voucher specimen numbers (IASST/BCCS/HNO112/2012, IASST/BCCS/HNO114/2012, IASST/BCCS/HNO115/2012) were deposited in Drug Discovery Laboratory, Institute of Advanced study in Science and Technology, Guwahati, Assam, India.

### Preparation of Extracts

The leaf, bark, and fruit of the plant was washed, chopped into small pieces and dried under shade. Dried samples were grounded to powder and extracted using 1000 ml methanol by maceration with continuous stirring for 3 days at room temperature (25 ± 2°C). This extraction was repeated three times. The extracts were filtered through Whatman filter paper to separate the solvent and marc. Final extracts were concentrated through vacuum evaporation (Buchi R-300, USA) at 45°C and all dried extracts were stored in tightly closed containers at -20°C until used for pharmacological testing (Sowemimo et al., 2015).

### Measurement of *In Vitro* Antioxidant Activity

#### DPPH Free Radical Scavenging Assay

2,2-Diphenyl-picrylhydrazyl free radical scavenging activity was determined by following (Sarwar et al., 2015). Briefly, 2.7 ml of 0.2 mM DPPH in methanol solution was added to 0.3 ml of the sample solutions. The reaction mixture was shaken and incubated at room temperature for 1 h and absorbance was measured at 517 nm. The radical scavenging activity was calculated by using following formula-

Scavenging⁢rate ={(As−Ai)/As}×100

Where As is the absorbance of pure DPPH and Ai is the absorbance of DPPH in the presence of various samples. In this assay BHT was used as standard.

#### Reducing Power Assay

The reducing power of the extracts was measured by the method of Oyaizu (1986). Briefly, various concentrations of 0.2 ml of samples were mixed with 2.5 ml phosphate buffer (0.2 M, pH 6.6) and 2.5 ml of 1% potassium ferricyanide. After incubation at 50°C for 20 min, 2.5 ml trichloroacetic acid (10%) was added and each reaction mixture was centrifuged at 100 rpm for 10 min. Then 2.5 ml supernatant was collected and mixed with 2.5 ml water and 0.5 ml ferric chloride (0.1%). The absorbance was measured at 700 nm. The increased absorbance of the reaction mixture indicated increased reducing power. BHT was used as a standard.

#### Lipid Peroxidation Inhibition Assay

Lipid peroxidation inhibition activity of the extracts was determined by thiobarbituric acid (TBA) assay of (Badmus et al., 2011). This assay is based on the reaction of the end product of lipid peroxidation, malondialdehyde (MDA) with TBA to form an adduct, a pink chromogen. Briefly, 0.1 ml of 100 μg/ml methanol extracts was added to 400 μl of double distilled water, 50 μL FeSO_4_ and 500 μl of 10% egg yolk. This reaction mixture was incubated for 30 min at 37°C, further 1.5 ml of 0.8% TBA in 1.1% SDS and 1.5 ml of 20% TCA was added. The reaction mixture was incubated for 1 h at 95°C, immediately cooled and 2 ml of butanol was added. The total reaction mixture was centrifuged at 3000 rpm for 5 min. The absorbance of the organic layer was measured at 532 nm. The percentage lipid peroxidation inhibition (LPI %) was calculated using the following formula:

LPI% =[(As−As)/AC]×100

*A*C = control absorbance, *A*S = sample absorbance

### *In Vitro* Anticancer Screening of GM Methanol Extracts

#### MTT Assay

Evaluation of cytotoxicity of the extracts on DLA cells was done by MTT assay. DLA cells were harvested in RPMI 1640 medium (10% FBS and antibiotic solution) and seeded in 96 well culture plate with different concentrations of the extracts. The culture plates were incubated for 3 h at 37°C and 5% CO_2_. After the incubation period, 10 μl MTT (4 mg/ml) was added to the wells and the plates were incubated in dark for 3 h. The formazan formed in wells was dissolved in 100 μl of DMSO and OD was measured at 570 nm.

#### Trypan Blue Assay

*In vitro* cytotoxic activity of the extracts on DLA cells was determined by trypan blue dye exclusion assay (Anjana et al., 2014). This dye is impermeable to live cells but enters the compromised membranes of dead cells. Briefly 1 × 10^6^ DLA cells in 1ml RPMI1640 media was incubated with different concentrations of the extracts for 3 h. After incubation cells were centrifuged and the pellet was resuspended in 0.5 ml of RPMI1640 media. 50 μl of cell suspension was mixed with equal amount of 0.4% trypan blue and the cells were counted using cell counter (Countess II FL, Life Technologies, USA). The percentage of cell cytotoxicity was determined as follows:

$$\%Cell\,cytotoxicity=100-\frac{Number\,of\,viable\,cells\,in\,the\,treated\,group}{Number\,of\,viable\,cells\,in\,the\,untreated\,control\,group}\times 100$$

### Cell Cytotoxicity by FACS Analysis

After incubation of 1 × 10^6^ DLA cells with different concentrations of the extracts for 3 h at 37°C, cells were washed with PBS and stained with 500 μl propidium iodide (PI) (1 mg/ml PI solution) to observe the percentage of cell death by FACS (BD Biosciences, USA) and the results were analyzed by using BD FACSuite^TM^.

### Effect of GF Methanol Extract on Apoptosis of DLA Cells

#### Caspase3 Assay

The levels of caspase3 in DLA cells on treatment with GF methanol extract was evaluated by using a caspase3 colorimetric assay kit (Catalog No: B3100) (R&D Systems, USA) (Xie et al., 2012). Briefly, 2 × 10^6^ DLA cells were incubated with 150 μg/ml and 250 μg/ml of GF extract for 3 h at 37°C. A control set without treatment was also kept in the same condition. After treatment the cells were washed with ice cold PBS and lysed with ice cold lysis buffer given in the kit. Assay procedure was followed according to the instructions given by the manufacturer. Absorbance of the plate was recorded at 405 nm in multimode reader (Thermo Scientific, USA).

### Effect of GF Methanol Extract on DLA Cell Morphology

#### Acridine Orange and Ethidium Bromide (AO/EB) Assay

Dalton’s Lymphoma cells treated with different doses of GF methanol extract were stained with AO/EB to observe morphological changes (Pajaniradje et al., 2014; Prasad and Koch, 2014). Briefly freshly aspirated DLA cells were washed twice with PBS (pH 7.4) and counted under inverted microscope. Viable cells were determined by staining with trypan blue 1 × 10^6^ cells were incubated with different concentrations of the extracts for 3 h at 37°C. After the incubation period, the cells were washed with PBS and stained with 20 μl of 100 μg AO/EB and observed under fluorescent inverted microscope.

### Scanning Electron Microscope (SEM) Analysis

After incubation of DLA cells with 150 μg/ml and 250 μg/ml of GF methanol extract, cells were observed under SEM (Carl Zeiss, SIGMAVP, Japan) to observe the cell morphology.

### Effect of GF Methanol Extract on DNA Fragmentation

DNA fragmentation assay was carried out by incubating 1 × 10^7^ cells with 50, 100, 150, and 200 μg/ml of GF methanol extract for 3 h in RPMI1640 at 37°C and 5% CO_2_. After incubation cells were washed twice with cold PBS and centrifuged, the cell pellet was lysed with lysis buffer (20 mM Tris HCl pH 8, 5 mM EDTA, 40 mM NaCl, and 1% SDS) on ice for 30 min. The lysate obtained was centrifuged and the supernatant was separated. To the supernatant 20 mg/ml proteinase K was added and the reaction mixture was incubated at 37°C for 2 h. The DNA was precipitated with absolute ethanol and 5 M NaCl overnight at -20°C. The DNA pellet was washed with 70% ethanol, air dried and dissolved in 1 × Tris-EDTA buffer. Electrophoresis was performed for the DNA obtained from each set in 1.8% agarose gel containing 0.5 μg/ml ethidium bromide. 200 bp DNA marker was run in the same gel (Prasad and Koch, 2014).

### Acute Toxicity Study

OECD guidelines were followed to conduct acute toxicity studies. Swiss albino mice of either sex were selected for this study. Animals were kept overnight fasting with free access to water and after 12 h, single oral dose of GF, GB, and GL at 2000 mg/kg body weight were administered to three animals. All the animals were observed for 14 days to check for mortality, if mortality was observed in 2 out of 3 animals, then the dose was identified as toxic dose. If mortality was observed in one animal, experiment was repeated again with same dose to confirm the toxic dose. If mortality observed again experiment was continued with low doses 300, 50, and 5 mg/kg body weight by following Ecobichon (1997).

### *In Vivo* Anticancer Activity of GF, GB, GL Methanol Extracts on DLA in Mouse

#### Animals

Male Swiss albino mouse weighing 22–25 g were procured from Pasteur Institute, Shillong and housed at Institute of Advanced Study in Science and Technology (IASST), Guwahati, Assam. Animals were acclimatized in polypropylene cages and fed with rodent pellet diet (Provimi Animal Nutrition India Pvt. Ltd., India) and water *ad libitum*. Temperature 22 ± 2°C, relative humidity 60–70%, and 12–12 h light–dark cycle was maintained throughout the experimental period and all experiments were performed during 9–14 h. The experimental protocol was approved (IASST/IAEC/2013–14/556) by the Institutional Animal Ethics Committee (IAEC) of IASST, Guwahati before starting the experiments and performed according to guidelines of Committee for Control and Supervision of Experimentation on Animals (CPCSEAs), Government of India.

#### Dalton’s Ascites Lymphoma Cells

Dalton’s ascites lymphoma (DLA) cells were kindly gifted by Dr. Surya Bali Prasad, NEHU, Department of Zoology, Shillong, India. Cells were maintained by intraperitoneal inoculation of 2 × 10^6^ cells/mouse.

#### Animal Grouping and Treatment Schedule

A total of seventy animals were divided into seven groups (*n* = 10). All the animals were injected with DLA cells (0.2 mL of 2 × 10^6^ cells/mouse) intraperitoneally, except the normal group, for the development of ascites tumor (Kuttan et al., 1988). The drug treatments were started subsequently by dissolving in 0.3% CMC and administered orally to the animals at 24 h intervals for 10 days.

Group I : Normal animals without inoculation with DLA cells.

Group II : DLA animals + Vehicle control (0.3% CMC)

Group III : DLA animals + 10 mg/ kg Cyclophosphamide

Group IV : DLA animals + 100 mg/kg GF methanol extract

Group V : DLA animals + 200 mg/kg GF methanol extract

Group VI : DLA animals + 200 mg/kg GL methanol extract

Group VII : DLA animals + 200 mg/Kg GB methanol extract

### Effect of the Extracts on Tumor Growth

The anticancer activity of the extracts were evaluated by analysis of different parameters like median survival time (MST) of each group (*n* = 10), % increase in life span (ILS) of the animals, changes in body weight and viable cell count. MST of each group was calculated with reference to the negative control group (Group-II) and anticancer activity of the drugs was determined by evaluating its ability to increase the life span of the DLA induced animals (Gupta et al., 2000). MST and % ILS were calculated by using the following formulae-

Mean survival time = [first Death + Last Death]/2

$$Increase\,of\,life\,span\,ILS\,(\%)=\frac{Mean\,survival\,of\,treated\,group\,\left(T\right)}{Mean\,survival\,of\,control\,untreated\,group\,\left(C\right)}\times 100$$

Where *T* = number of days treated animals survived *C* = number of days control animals survived

The survival curve was plotted using Kaplein–Meier method (Ghosh and Maiti, 2007; Kant et al., 2014) (**Figure 3**).

#### Hematological Parameters

At the end of experimental period, animals were sacrificed and blood was collected in EDTA coated tubes for estimation of red blood cells (RBC), white blood cell (WBC) and hemoglobin (Hb) by standard procedures (Rajesh et al., 2011).

#### Biochemical Parameters

Twenty 4 h after the last drug treatment, animals were sacrificed. Blood was collected immediately in tubes without anticoagulant and centrifuged at 3000*g* at 4°C for 10 min to separate the serum. Blood free serum was used for evaluating biochemical parameters such AST, ALT, and ALP levels (Priya et al., 2011) by using commercially available kits (Accurex, India) according to manufacturer’s instructions.

#### Neo Vascularisation

After sacrificing the animals on the 10th day the peritoneum of the mice were removed and the inner lining of the peritoneum were observed for angiogenesis. Photographs of the peritoneal linings were captured (Augustine et al., 2014).

#### Histopathological Analysis

Liver tissues were immediately fixed in 10% buffered formalin, embedded in paraffin, processed by means of routine histological techniques and stained with haematoxylin and eosin (Priya et al., 2011) to observe the pathological changes under light contrast microscope (10X).

### Effect of GB, GF, and GL Methanol Extracts on Dalton’s Lymphoma Cell Death

Swiss albino mice of weight 25 ± 2 g were divided into five groups of six animals in each group. 1 × 10^6^ DLA cells injected intraperitoneally to the animals for development of DLA ascites tumor. After 8 days of tumor development, drug was administered intraperitoneally for 2 days as follows-

Group-I : DLA mouse + Vehicle treatment (0.3% CMC)

Group-II : DLA mouse +GB methanol extract (200 mg/kg)

Group-III : DLA mouse + GF methanol extract (200 mg/kg)

Group-IV : DLA mouse + GL methanol extract (200 mg/kg)

Group-V : DLA mouse + Cyclophosphamide (10 mg/kg)

Four hours after the last drug treatment, DLA cells were aspirated from the animals and washed with PBS, incubated in 500 μL PI (1 mg/ml PI solution) for 5 mins and observed in FACS for detection of cell death. Results were analyzed by using BD FACSuite^TM^. Total experimental procedure was repeated thrice to confirm the results accurately. Fold change in % cell death was calculated by the formula-

$$Fold\,change\,=\frac{\%cell\,death\,in\,treated\,group}{\%cell\,death\,in\,untreated\,(control)group}$$

The PI stained cells were also observed under fluorescent microscope to mark the morphological changes in nuclei of DLA cells aspirated from treated groups in comparison to the vehicle control group.

### Statistical Analysis

All the results are expressed in mean ± SD. Experimental data was analyzed by one way ANOVA followed by Tukey’s multiple comparison test was used to compare the different parameters between the groups. A *P* < 0.05 was considered as significant. The survival study was analyzed by Kaplein–Meier method. All the statistical analysis was done by using the GraphPad Prism 6 software.

## Results

### *In vitro* Antioxidant Assays

#### GM Extracts Scavenge DPPH Free Radicals

**FIGURE 1:**
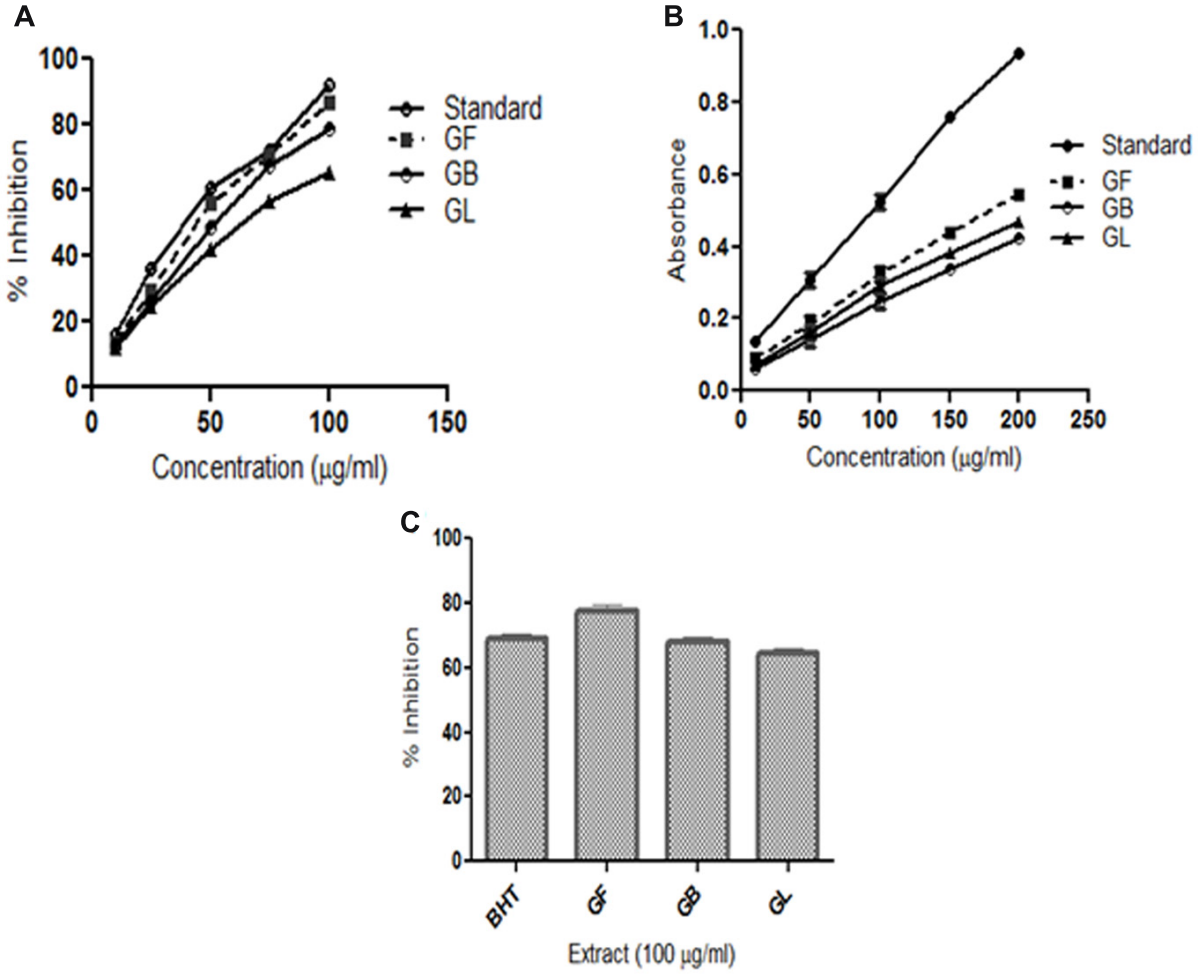
***In vitro* antioxidant assays **(A)** DPPH radical scavenging activity **(B)** Reducing power activity **(C)** Lipid peroxidation inhibition activity of methanol extracts of *G. morella* fruit (GF), bark (GB), leaf (GL), and standard BHT**.

2,2-Diphenyl-picrylhydrazyl is a free radical compound and it is accepted in estimating the scavenging activity of antioxidants. The DPPH scavenging activities of different extracts are presented in **Figure 1A.** The methanol extract of *G. morella* fruit, leaf and bark were screened for *in vitro* antioxidant activity at different concentrations 10, 25, 50, 75, and 100 (μg/ml). From **Figure 1A**, the methanol extracts of GM fruit, leaves and bark have shown high DPPH scavenging activity in a dose dependent manner. GF extract showed the highest DPPH free radical scavenging activity in comparison to the GB and GL.

#### Reducing Power Ability of GM Extracts

Reducing power of a substance is the measure of the antioxidant capacity of the substance. (Meir et al., 1995). In this assay, the reducing power of the extracts was determined by measuring the potentiality of the extracts in reducing Fe^3+^ to Fe^2+^. On formation of Fe^2+^ the solution turns prussian blue and the intensity of color formation is directly proportional to Fe^2+^ formation. The OD of the solutions at 700 nm thus represents the reducing power of the extracts. From (**Figure 1B**) the reducing power of the extract increases on increase in concentration. The order of reductive potential is thus BHT > GF > GL > GB.

#### Lipid Peroxidation Inhibition Activity

Lipid peroxidation inhibition ability of different extracts of *G. morella* is given in (**Figure 1C**). Among all the extracts GF methanol extract showed the highest inhibition at 100 μg/ml which was even higher than the standard BHT.

#### Treatment with GF Extract Induces Death of DLA Cells in *in vitro* Condition

**FIGURE 2:**
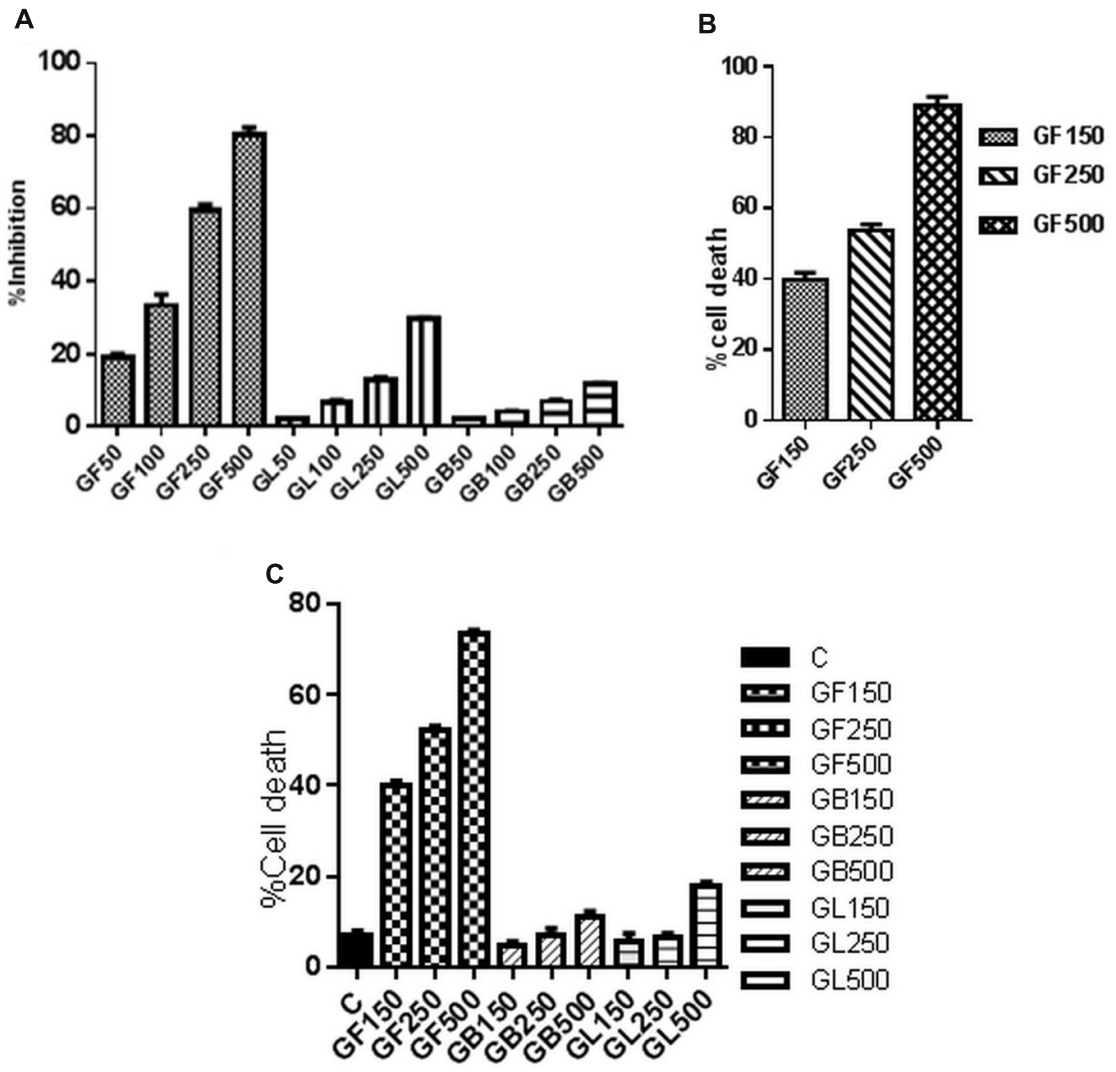
***In vitro* cell viability of DLA cells monitored by **(A)** MTT assay and **(C)** FACS analysis after 3 h treatment of DLA cells with different concentrations of GF, GB, and GL and **(B)** Trypan blue dye exclusion assay of DLA cells on treatment with GF at different concentrations for 3 h.** Data are shown as % cell death for FACS and trypan blue assay and % inhibition of cell proliferation for MTT assay. % cell death and % inhibition of cell proliferation are calculated by comparing with the untreated control group. All the results are expressed in mean ± SD (*n* = 3).

The GF extract has shown cytotoxic activity in a dose dependent manner on DLA cells as observed from MTT assay, trypan blue dye exclusion method and FACS analysis (**Figure 2**). In FACS analysis (**Figure 2C**), it was observed that the GF extract exerted the highest cytotoxic impact on DLA cells. GF methanol extract 150, 250, and 500 μg/ml caused 45.7, 63.2, and 73.9% cell death, respectively. From the MTT assay the IC50 of GF on DLA cells was found to be 250 μg/ml (**Figure 2A**). From trypan blue dye exclusion method it was observed that GF methanol extract showed dose dependent increase in % cell death of DLA cells (**Figure 2B**).

#### GF Extract Induces Apoptosis of DLA Cells

**FIGURE 3:**
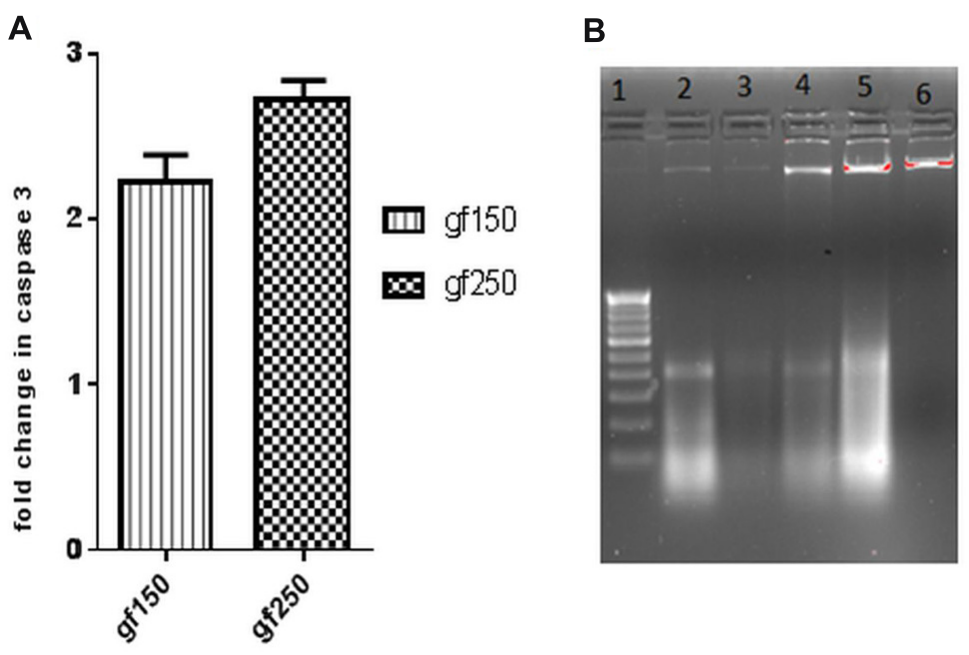
***In vitro* apoptotic effect of GF on DLA cells was determined by **(A)** fold change in level of caspase3 of DLA cells on treatment with GF (150 and 250 μg/ml) for 3 h.** Fold change is calculated by comparing with untreated DLA cells. All the results are expressed in mean ± SD (*n* = 3). **(B)** DNA fragmentation in different doses of GF treated DLA cells. 10 μg DNA from each treatment groups and untreated group was loaded in each lane and subjected to1.8% agarose gel electrophoresis followed by detection of EtBr stained DNA bands in UV transilluminator. The photograph is a representative of three repeats. Here lane1:200 bp DNA ladder, lane2, 3, 4, and 5 represents DNA of DLA cells treated with 50, 100, 150, and 200 μg/ml GF for 3 h. lane6: DNA of untreated DLA cells.

GF methanol extract significantly increased the caspase3 activity in DLA cells. Caspase cascade activation plays an important role in apoptosis. To determine the role of caspases in GF extract induced apoptosis, the activity of caspase3 was investigated. Treatment of DLA cells with GF at 150 μg/ml and 250 μg/ml for 3 h dose dependently increased the fold change in caspase3 level relative to the untreated DLA cells. Treatment with 150 μg/ml and 250 μg/ml GF methanol extract resulted in 2.35 and 2.72-fold increase (*p* < 0.05), respectively, in caspase3 level (**Figure 3A**) of DLA cells.

**FIGURE 4:**
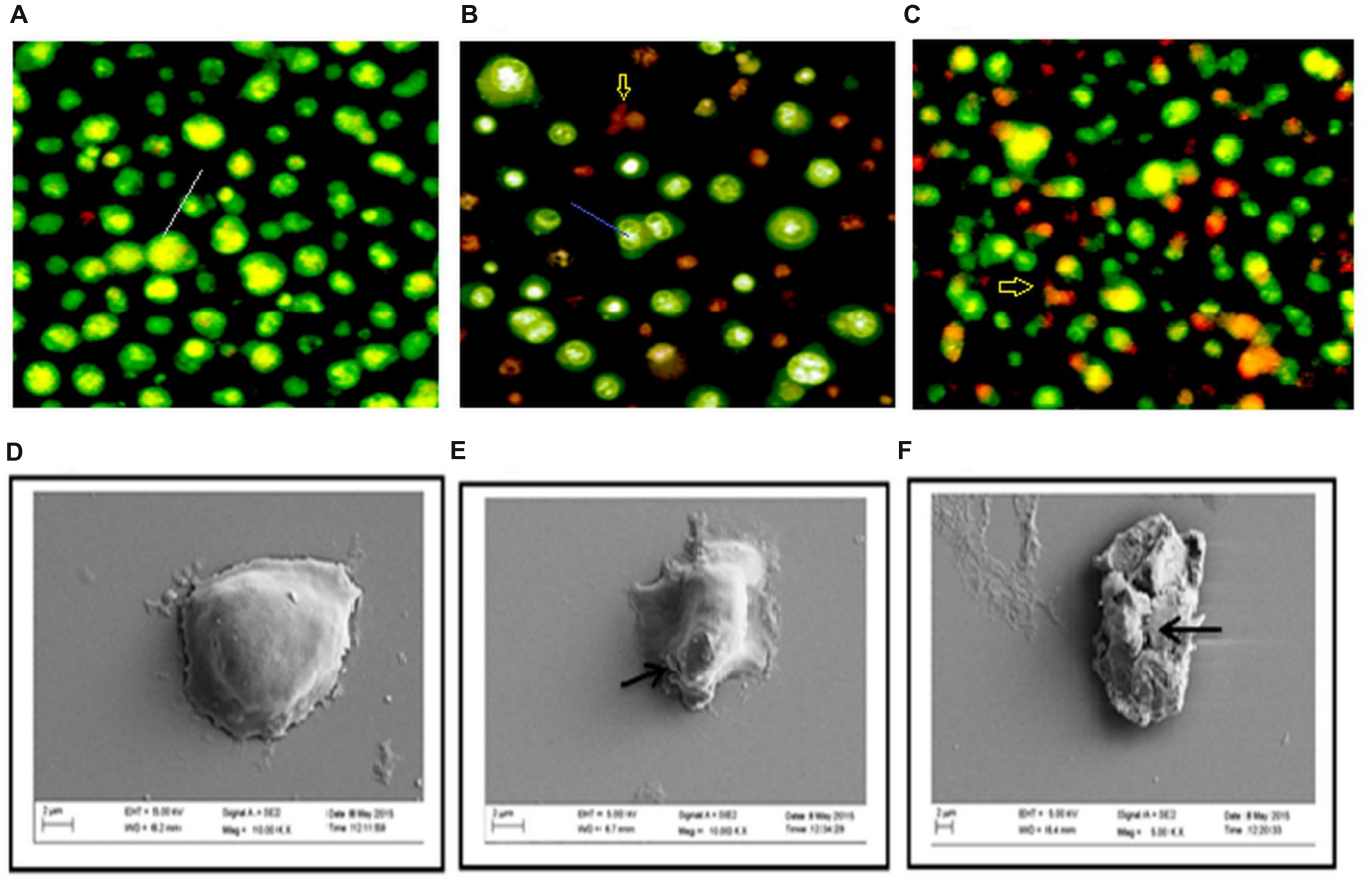
**Changes in morphology of DLA cell on treatment with GF was determine by staining untreated and GF treated DLA with AO/EtBr **(A–C)**.** Here **(A)** is the untreated DLA cells **(B)** DLA cells treated with 150 μg/ml GF and **(C)** DLA cells treated with 250 μg/ml GF for 3 h. The white arrows are pointing to normal live cells, blue arrows are directing toward cells having shrinked nucleus representing early apoptosis and red arrows are pointing toward cells containing yellowish orange nuclei representing apoptotic cells. **(D–F)** Scanning electron microscope images of DLA cells afters treatment with GF 150 and 250 μg/ml for 3 h. **(D)** Control DLA cells. **(E)** DLA cells treated with GF 150 μg/ml and **(F)** DLA cells treated with GF 250 μg/ml for 3 h. Untreated DLA are observed to have smooth surface but the DLA cells treated with GF extract are shown have many ruptures on the surface.

Acridine orange/ethidium bromide staining was done to observe the morphological changes of the DLA cells upon treatment with the extracts for 3 h. The cells showing green fluorescence with intact green nucleus represents live cells in both control (**Figure 4A**) and treatment groups (**Figures 4B,C**). On treatment with GF 150 and 250 μg/ml distinct morphological changes were observed in the DLA cells such as cell shrinkage, blebbing, and more number of orange/red cells (apoptotic cells). SEM images of the GF treated DLA cells also displayed the appearance of membrane blebbing which signified the initiation of apoptosis (**Figures 4E,F**) which was completely absent in control (**Figure 4D**). To confirm the induction of apoptosis by GF methanol extract on DLA cells DNA fragmentation assay was done. DNA of the treated DLA cells was observed to display laddering pattern, in contrast the untreated cells showed intact DNA (**Figure 3B**). On late apoptosis stage, DNA fragments at an interval of 180–200 bp and DNA band appear like ladder in the gel. So we could confirm that treatment with GF conferred apoptotic induction and death in the DLA cells.

### Acute Toxicity Study

Acute toxicity study was conducted to determine the toxicity level of the drug. All the extracts were given at a dose of 2000 mg/kg to the mice and observed for 14 days. None of the extracts caused any change in general behavior or lethality to the mice when observed for 14 days. So 1/10th of non-lethal dose was selected for *in vivo* anticancer assay. From *in vitro* assays it was observed that GF methanol extract was highly antioxidant and cytotoxic than GB and GL methanol extracts. Hence two doses were selected for GF methanol extract (100 and 200 mg/kg) and only one dose was selected for the remaining two extracts (200 mg/kg).

### Effect of the Extracts on Tumor Growth

GF and GL methanol extracts were significantly cytotoxic to DLA cells, whereas GB methanol extract did not produce significant activity (**Table 1**).

**Table 1:** Effect of different drug treatments on *In vivo* tumor growth.

S.no	Treatment	Viable tumor cells count (10^5^ Cells/ml)	Weight change (gm)	MST	% ILS
1	DLA	558 ± 7.45	13.5 ± 0.96	12.2 ± 1.28	-
2	DLA + CYLP (10 mg/kg)	9.3 ± 1.22ˆ***	5.5 ± 0.02ˆ***	28.4 ± 2.12	132.7
3	DLA + GF (100 mg/kg)	62.9 ± 4.17ˆ***	8.2 ± 0.74ˆ***	16.8 ± 1.72	37.7
4	DLA + GF (200 mg/kg)	22 ± 2.72ˆ***	6.3 ± 0.57ˆ***	20.3 ± 1.61	66
5	DLA + GB (200 mg/kg)	105.6 ± 4.94ˆ***	7.3 ± 0.42ˆ***	14.5 ± 1.37	18.85
6	DLA + GL (200 mg/kg)	44.8 ± 3.47ˆ***	5.6 ± 0.16ˆ***	18.4 ± 1.98	50.8


All the results were expressed in mean ± SD (*n* = 10). ***P ≤ 0.001 in comparison of drug treated DLA mice with untreated DLA mice. DLA, Dalton’s lymphoma; CYLP, Cyclophosphamide; GF, *G. morella* fruit methanol extract; GB, *G. morella* bark methanol extract; GL, *G. morella* leaf methanol extract.

The weight of the DLA induced mice gradually increases with passage of time due to accumulation of ascitic fluid in the belly. Hence change in weight is considered as a parameter to determine the intensity of the disease. From **Table 1**, it was observed that the GF and GL methanol extracts at 200 mg/kg caused no significant increase in body weight of the animal, i.e., of about 6.3 and 5.6 gm, respectively, while in the untreated group of animals the increase in weight was about 13.5 gm (**Figures 7B1,2**).

**FIGURE 5:**
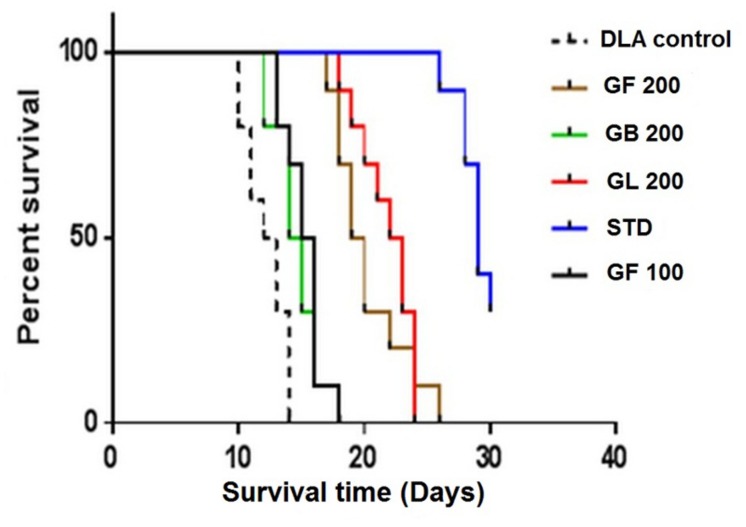
**Effect of the *G. morella* extracts on longevity of DLA induced mice is represented by Kaplan–Meir curve**.

Treatment with all the extracts GF, GB, and GL increased the life span of DLA induced mice. GF methanol extract at 200 mg/kg increased the life span of the mice to 66% whereas the standard drug treated group had a percentage increase ILS of about 132.7 with respect to the untreated group of animals. From the mean survival time (MST) data also it was evident that the GF could increase the MST of the DLA induced mice in a dose dependent manner. As observed from the Kaplan–Meier curve (**Figure 5**) GF methanol extract displayed the highest activity in increasing the longevity of DLA induced mice.

### Effect of Drug Treatments on Hematological Parameter

It was observed that the level of WBC severely raised up for the DLA induced untreated group of animals whereas the RBC count and hemoglobin count of these group drastically dropped down as compared to normal animals (**Table 2**). Interestingly, GF and GL extracts could restore the hematological parameters of the DLA induced mice. The GF and GL extract at 200 mg/kg could increase the RBC count to 3.67 ± 0.21^$$$^ and 3.81 ± 0.20^$$$^ (Cells/ml × 10^6^), respectively, and lowered the WBC count to 14.20 ± 0.21^$$$^ and 14.81 ± 0.18^$$$^ (Cells/ml × 10^3^), respectively.

**Table 2:** Effect of different drug treatments on hematological parameters.

S. no	Treatment	RBC (Cells/ml X 10^6^)	WBC (Cells/ml X 10^3^)	Hb g/dl
**1**	Normal	5.19 ± 0.21	11.23 ± 0.28	14.66 ± 0.20
**2**	DLA control	2.35 ± 0.18^∗∗∗^	17.47 ± 0.31^∗∗∗^	7.78 ± 0.13^∗∗∗^
**3**	DLA + CYLP (10 mg/kg)	3.92 ± 0.22^$$$^	13.43 ± 0.26^$$$^	12.36 ± 0.18^$$$^
**4**	DLA + GF (100 mg/kg)	3.27 ± 0.18^$$$^	15.37 ± 0.19^$$$^	10.35 ± 0.17^$$$^
**5**	DLA + GF (200 mg/kg)	3.67 ± 0.21^$$$^	14.20 ± 0.21^$$$^	11.88 ± 0.21^$$$^
**6**	DLA + GB (200 mg/kg)	2.71 ± 0.23^$$^	16.33 ± 0.22^$$$^	8.05 ± 0.14^$$^
**7**	DLA + GL (200 mg/kg)	3.81 ± 0.20^$$$^	14.81 ± 0.18^$$$^	11.28 ± 0.21^$$$^


All the results were expressed in mean ± SD (*n* = 10). ***P ≤ 0.001 comparison of DLA untreated mice with normal mice, $$$ *P* ≤ 0.001 and $$ *P* ≤ 0.01 comparison of drug treated DLA mice with DLA untreated mice. DLA, Dalton’s lymphoma; CYLP, Cyclophosphamide; GF, *G. morella* fruit methanol extract; GB, *G. morella* bark methanol extract; GL, *G. morella* leaf methanol extract.

### Effect of Drug Treatment on Biochemical Parameters

Inoculation of DLA cells in mice caused significant increase in levels of enzymes such as AST, ALT, and ALP. It was evident that the treatment with the standard cyclophosphamide could bring back the serum enzyme levels to the normal range (**Table 3**). The GF (200 mg/kg) treated group of animals also had a comparable range of enzyme levels with normal animals. The effect of the extracts in restoring the enzyme levels was significant.

**Table 3:** Effect of different drug treatments on serum biochemical parameters.

S.no	Treatment	AST	ALT	ALP
**1**	Control	41.14 ± 2.36	34.18 ± 1.79	128.52 ± 2.21
**2**	DLA	87.17 ± 3.11^∗∗∗^	57.19 ± 2.41^∗∗∗^	247.35 ± 3.62^∗∗∗^
**3**	DLA + CYLP (10 mg/kg)	49.47 ± 2.62^$$$^	42.33 ± 1.87^$$$^	147.89 ± 1.68^$$$^
**4**	DLA + GF (100 mg/kg)	67.47 ± 3.38^$$$^	49.51 ± 2.64^$$$^	202.91 ± 2.48^$$$^
**5**	DLA + GF (200 mg/kg)	56.92 ± 2.76^$$$^	44.58 ± 3.11^$$$^	177.77 ± 3.15^$$$^
**6**	DLA + GB (200 mg/kg)	73.92 ± 3.61^$$^	52.71 ± 2.86^$$^	231.09 ± 4.23^$^
**7**	DLA + GL (200 mg/kg)	61.46 ± 1.94^$$$^	47.96 ± 1.82^$$$^	188.24 ± 2.27^$$$^


All the results were expressed in mean ± SD (*n* = 10). ***P ≤ 0.001 comparison of DLA untreated mice with normal mice, $$$ *P* ≤ 0.00, $$ *P* ≤ 0.01 and $ *P* ≤ 0.05 comparison of drug treated DLA mice with DLA untreated mice. DLA, Dalton’s lymphoma; CYLP, Cyclophosphamide; GF, *G. morella* fruit methanol extract; GB, *G. morella* bark methanol extract; GL, *G. morella* leaf methanol extract.

### Effect of Drug Treatment on Neo Vascularisation

**FIGURE 6:**
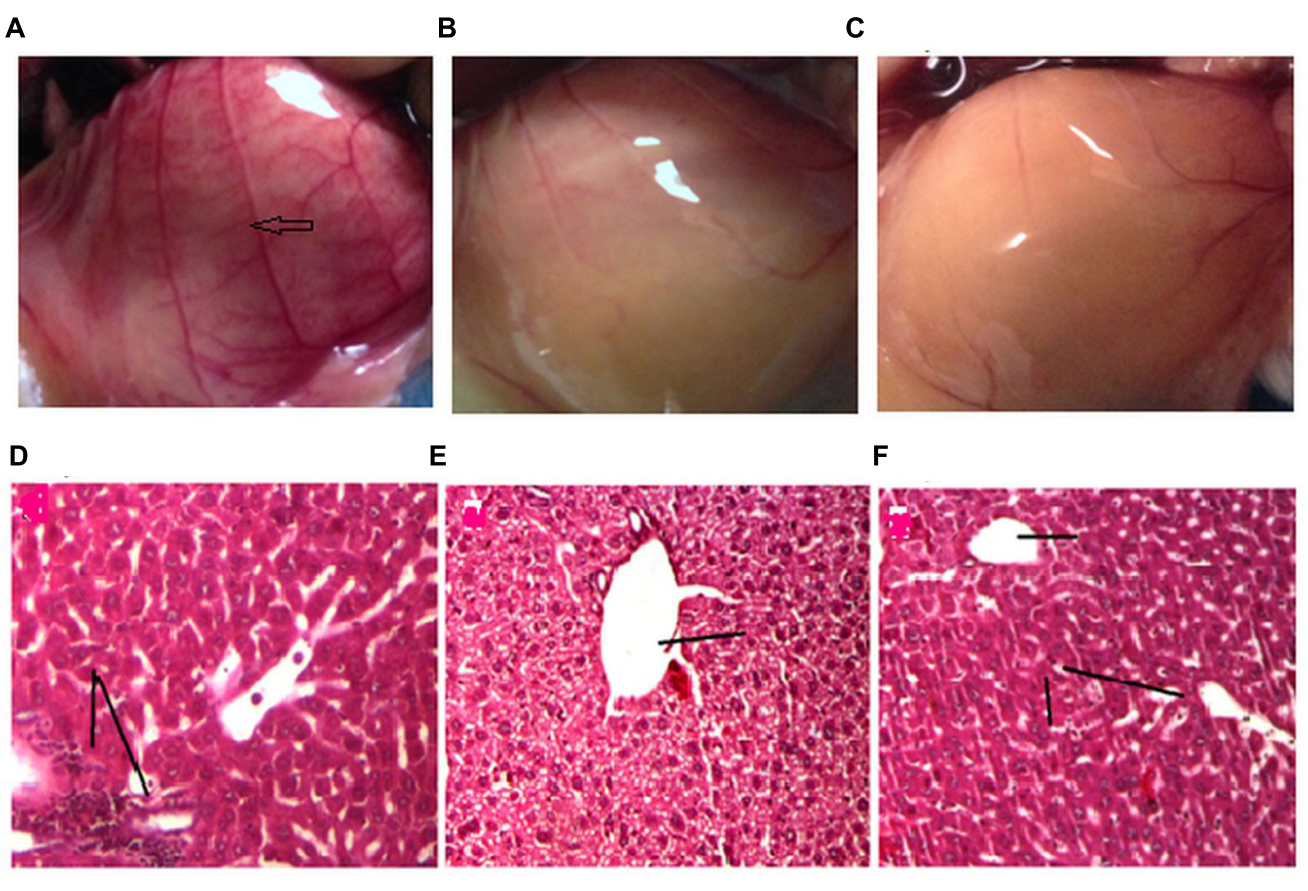
**Effect of drug treatment on neo vascularisation **(A–C)** and liver histology **(D–E)**.** The figures **(A–C)** represent the inner peritoneum lining of untreated, standard treated and GF 200 mg/kg treated DLA induced animals, respectively. The blue arrow is indicating new vasculature in the peritoneum of untreated DLA induced mice. The GF treated and Standard treated animals doesn’t show any significant angiogenesis. **(D–F)** is the liver histology of DLA induced untreated, standard treated and GF200 mg/kg treated animals.

The peritoneum of the DLA induced animals untreated animals (control) (**Figure 6A**) showed greater number of blood vessels than the treatment groups (**Figures 6B,C**). From the critical observation of the photographs it can be concluded that treatment of DLA animals with GF methanol extract at 200 mg/kg and cyclophosphamide (10 mg/kg) significantly inhibited neovascularisation.

### Effect of Drug Treatments on Liver Histopathology

Liver tissue of DLA bearing mice in vehicle treated group showed degenerative hepatocytes with periportal inflammation, inflammatory infiltrate is composed mainly of lymphocytes (**Figure 6D**). DLA animals treated with standard drug (**Figure 6E**) and GF methanol extract (**Figure 6F**) showed protection against hepatic damage, even though in case of extract treated group mild lymphocyte infiltration was observed.

### *In Vivo* Apoptotic Induction of DLA Cells on Treatment with GF

**FIGURE 7:**
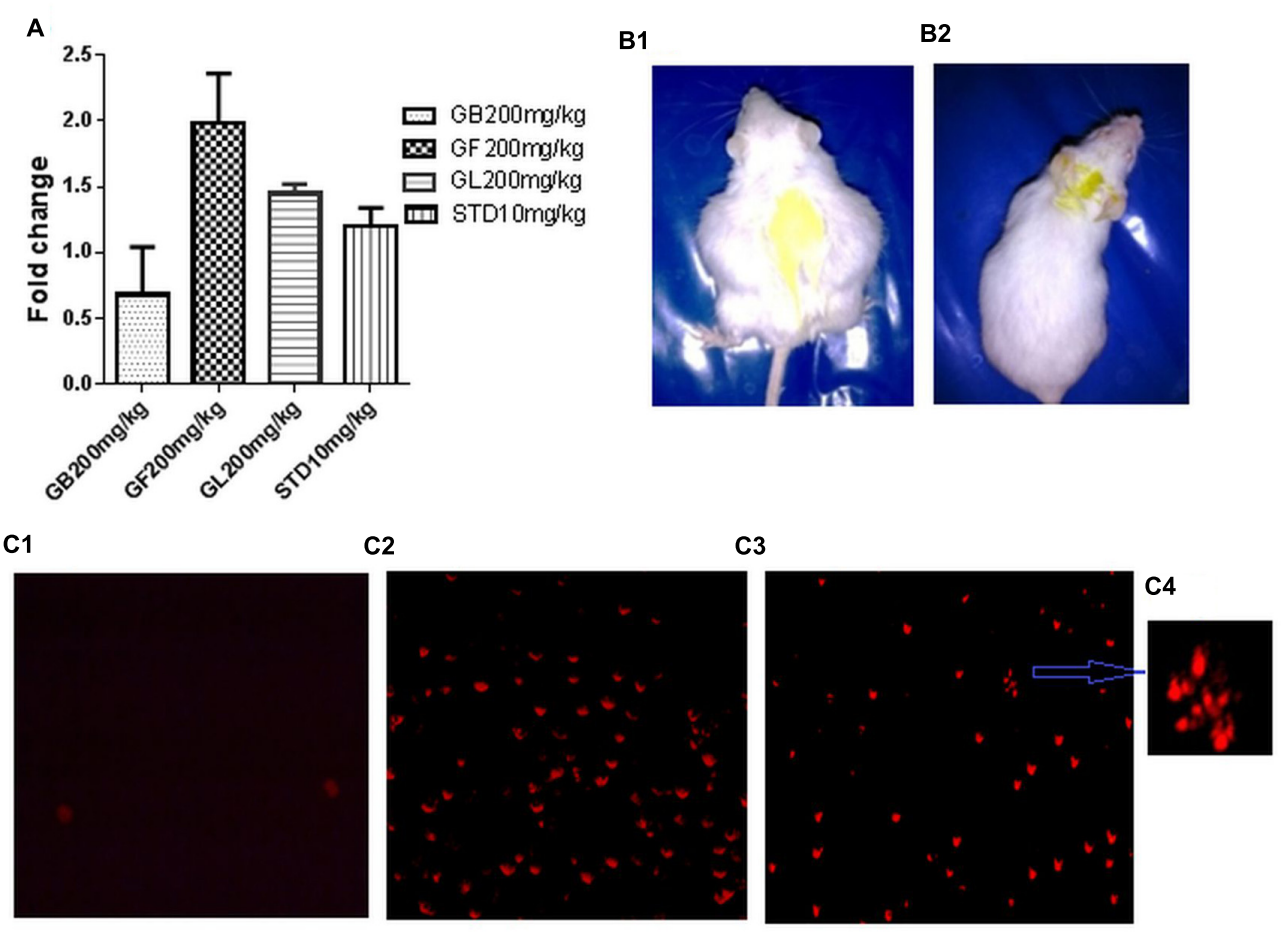
**(A)** Fold change in *in vivo* DLA cell death measured by FACS analysis of DLA cells aspirated from the different *in vivo* experimental group after staining with PI. **(B1,2)** are pictures of DLA induced untreated and GF 200 mg/kg treated mice after 10 days of drug treatment. **(C1–3)** represents the fluorescent microscope images of DLA cells aspirated from the untreated, standard treated and GF200 mg/kg treated *in vivo* experimental groups after staining with PI. **(C4)** is a zoomed image from **(C3)** showing apoptotic morphology of GF 200 mg/kg treated group.

It was observed that GF methanol extract at 200 mg/kg caused the maximum increase in fold change of % cell death of approximately 1.98. Whereas in GB, GL methanol extracts and standard drug treated groups, the fold change of about 0.68, 1.2, and 1.76 was observed (**Figure 7A**).

To confirm the induction of apoptotic cell death in DLA cells, PI staining was done to the cells from different treatment groups. Results showed that DLA cells treated with GF methanol extract at 200 mg/kg showed prominent apoptotic morphology (**Figures 7C3,4**) This observation is comparable with standard treated group (**Figure 7C2**), whereas in untreated cells negligible cell death was observed (**Figure 7C1**).

## Discussion

The results of the present study reports the dose dependent anticancer activity of *G. morella* fruit extract on DLA induced ascites tumor models. The present study also reveals the presence of strong antioxidant compounds in the methanol extracts of *G. morella* fruit, bark and leaf, which was demonstrated by *in vitro* DPPH scavenging, reducing power, and lipid peroxidation inhibition assays. The experimental results showed that GF extract possessed strong antioxidant activity than GB and GL extracts in all the experiments conducted. IC 50 value of GF in DPPH radical scavenging assay was found to be 50 μg/ml, which is very low in comparison to the previous report (Gogoi et al., 2012), where they recorded 303.63 ± 5.34 μg/ml. This enhancement in activity was might be due to the change in geographical location, season, and time of collection of fruit samples. Free radicals are formed naturally in the body and play important role in cellular processes. However, at higher concentrations, it can cause hazardous damage to the cellular components, like DNA, protein, and cell membranes. Damage especially to the DNA can trigger the development of cancer (Li et al., 2013b). Studies have revealed that antioxidants have the ability to save the cells from the damaging effects of free radicals. Dietary antioxidants, such as lycopene, beta carotene, alpha tocopherol are known to be used for cancer chemoprevention and therapy (Gloria et al., 2014; Kim and Kim, 2015).

Several studies have been reported previously, which indicate that the molecules possessing antioxidant activities can possibly slow or prevent the development of cancer (Islam et al., 2013). The results of antioxidant assays therefore inspired us to evaluate the effects of the GM extracts on cancer cells. The significant anti-proliferative activity of GF with an IC50 of 250 μg/ml demonstrates its anticancer potential, which was also observed in FACS analysis where methanol extract of GF at 250 μg/ml caused 63.2% cell death. Treatment with this extract also resulted in loss of membrane integrity of DLA cells thereby making trypan blue dye permeable through the membrane. Similarly, Prasad and Koch (2014) reported the IC50 of *Dendrobium formosum* on DLA cells to be 350 μg/ml.

The subsequent study to understand the underlying molecular mechanism of GF on DLA revealed that GF induces apoptosis in DLA cells by activation of caspase cascade and fragmentation of DNA. Apoptosis is one of the key targets of chemotherapeutic drugs in cancer therapy. It is a programmed cell death in which the dead cells are removed without causing inflammation and damage to the nearby cells. Apoptosis is characterized by membrane blebbing, cell shrinkage, chromosomal DNA fragmentation (Prasad and Koch, 2014). Presence of membrane blebbing and yellowish/orange condensed chromatin in DLA cells after treatment with GF extract at different doses for 3 h clearly indicating the apoptotic properties of GF extract in DLA cells. The SEM images also proved cell membrane blebbing, which indicates apoptosis of the DLA cells upon treatment with GF. One of the major biochemical hallmarks of apoptosis is the activation of caspase cascade, in which series of cysteinyl aspartic acid specific proteases get activated by cleavage of their zymogen form and this leads to the activation of two main pathways of apoptosis: the extrinsic death receptor mediated and intrinsic mitochondrial mediated apoptosis. Caspase 3 and 7, which are also known as effector caspases gets activated in both the pathways, while caspase 8 and caspase 9 (initiator caspases) are involved only in extrinsic and intrinsic pathways, respectively, (Patil et al., 2010). Caspase 3 activation induces PARP cleavage, DNA fragmentation and ultimately kills the cells by apoptosis.

We further confirmed the anticancer activity of GM by treatment of DLA induced mice with GF, GB, and GL methanol extracts. In judgment of anticancer activity of a drug, the prolongation ILS of the animals and the decrease of leukemic cells from blood is considered as reliable criteria (Gupta et al., 2004). More interestingly, for the first time, we found that the DLA bearing mice administered with 100 mg/kg and 200 mg/kg of GF prolonged the life span of the animals significantly than the untreated group. Decrease in viable DLA cell count in GF treated group of animals compared to the untreated group indicates that the test drug is having *in vivo* anti proliferative activity on DLA cells. Similar studies have been reported Augustine et al. (2014), where they found that the ethyl acetate fraction of *Leucas aspera* significantly reduced the viable DLA cell count on treatment of DLA induced mice with it. The anticancer property of the *G. morella* can also be attributed by its *in vitro* cytotoxic property as evidenced by its *in vitro* activity (Loganayaki and Manian, 2012).

The tumor control group was marked by increase in WBC and decrease in RBC count. It is found from our results that treatment with GF extract could restore the hematological parameters in the GF treated groups. In this case it can be postulated that due to progression of cancer, WBC levels increased in the DLA induced animals. On treatment with the GF extract, the WBC and RBC levels were significantly restored.

Several studies have reported that on induction of cancer in experimental animals, cause damage to the vital organs, such as liver and kidney. ALP levels rises in plasma on obstruction of large bile duct, intra hepatic cholestasis or infiltrative diseases of the liver (Harada et al., 1986). In the present study it was observed that DLA bearing mice showed higher levels of serum AST, ALT, and ALP levels and severe damage to the hepatocytes. GF extract significantly restored the serum enzyme levels and protects the hepatocytes of DLA bearing mice.

Tumor cells growing in *in vivo* condition are in a hypoxic state (Koiri et al., 2009). This activates hypoxia inducible factor 1α (HIF-1α) and this may lead to initiation of angiogenesis by activation of downstream genes, such as VEGF (Zhao et al., 2015). There is evidence that standard drug such as cyclophosphamide has antiangiogenic activity in cancer induced animal model (Colleoni et al., 2002). GF (200 mg/kg) significantly inhibited the growth of new blood vessels in the tumor area. Thus, it can be hypothesized that GF extract may have inhibited hypoxia inducible factors or in the downstream of the pathway, which ultimately led to successful inhibition of angiogenesis. Further molecular study in this regard may lead to successful identification of the exact mechanism of action of GF on angiogenesis.

## Conclusion

The current study could clearly reflect the highly antioxidant and anticancer efficacy of methanol extract of fruits of *G. morella*. Its ability to increase the longevity of DLA induced mice and restore their hematological and biochemical parameters was quite significant. This study also suggests that the cytotoxic effect of GF on DLA is due to induction of caspases and DNA fragmentation, which finally led to apoptosis of the DLA cells. In addition the anti angiogenic evaluation of GF on DLA suggests the inhibition of angiogenic mediators like VEGF. Considering the findings of this study, it seems quite possible that *G. morella* fruits may lead to the development of new anticancer and anti angiogenic drug in the near future.

## Author Contributions

Miss. Choudhury is the main author of this research work, including study design, literature survey, plant collection, extraction and experimental set up. She performed all the *in vitro* and *in vivo* assays including molecular biology work and all the biochemical assays. Mr. Kandimalla contributed toward the animal experimentation including experimental setup, animal handling, maintenance, dosage administration, biological fluids collection etc. Miss. Monisha contributed in performing FACS analysis Prof. Bharali contributed toward study design and traditional knowledge based literature review Dr. Kunnumakkara contributed in assay design, experimental setup, supervision, and results assessment. Dr. Kalita performed and analyzed the histopathology data. Prof. Kotoky contributed in supervision of the work and compiling all the results.

## Conflict of Interest Statement

The authors declare that the research was conducted in the absence of any commercial or financial relationships that could be construed as a potential conflict of interest.
